# L1 variation and L2 acquisition: L1 German /eː/-/ɛː/ overlap and its effect on the acquisition of L2 English /ɛ/-/æ/

**DOI:** 10.3389/fpsyg.2023.1133859

**Published:** 2023-06-28

**Authors:** Marcel Schlechtweg, Jörg Peters, Marina Frank

**Affiliations:** ^1^Institute for English and American Studies, Carl von Ossietzky Universität Oldenburg, Oldenburg, Germany; ^2^Institute for German Studies, Carl von Ossietzky Universität Oldenburg, Oldenburg, Germany; ^3^Research Center Deutscher Sprachatlas, Philipps-Universität Marburg, Marburg, Germany

**Keywords:** acquisition, L1 German, L2 English, sociophonetics, vowels, variation

## Abstract

A person’s first language (L1) affects the way they acquire speech in a second language (L2). However, we know relatively little about the role different varieties of the L1 play in the acquisition of L2 speech. This study focuses on German (L1) learners of English (L2) and asks whether the degree to which German speakers distinguish between the two vowels /eː/ and /ɛː/ in their L1 has an impact on how well these individuals identify /æ/ and discriminate between the two English vowels /ɛ/ and /æ/. These two English vowels differ in both vowel quality and duration (/æ/ is longer than /ɛ/). We report on an identification and a discrimination experiment. In the first study, participants heard a sound file and were asked to indicate whether they heard “pen” or “pan” (or “pedal” or “paddle”). The stimuli differed from each other in terms of both vowel quality (11 steps on a spectral continuum from an extreme /æ/ to an extreme /ɛ/) and duration (short, middle, long). In the second study, participants had to signal whether two sound files they were exposed to differed from each other. We modeled the percentage of /æ/ (“pan,” “paddle”) selection (identification task only, binomial logistic regression), accuracy (discrimination task only, binomial logistic regression), and reaction time (identification and discrimination tasks, linear mixed effects models) by implementing the German Pillai score as a measure of vowel overlap in our analysis. Each participant has an individual Pillai score, which ranges from 0 (= merger of L1 German /eː/ and /ɛː/) to 1 (=maintenance of the contrast between L1 German /eː/ and /ɛː/) and had been established, prior to the perception experiments reported here, in a production study. Although the findings from the discrimination study remain inconclusive, the results from the identification test support the hypothesis that maintaining the vowel contrast in the L1 German leads to a more native-like identification of /æ/ in L2 English. We conclude that sociolinguistic variation in someone’s L1 can affect L2 acquisition.

## 1. Introduction

A person’s first language (L1) affects how they acquire a second language (L2), for instance, how this person perceives speech in the L2. This idea is central to several well-established models of L2 speech, such as the Speech Learning Model (SLM, Flege, 1995), the Perceptual Assimilation Model for the L2 (PAM-L2, Best and Tyler, 2007), and the Revised Speech Learning Model (SLM-r, Flege and Bohn, 2021; for discussion, see also Mora et al., 2022). According to these conceptualizations of the L2, the L1 can shape L2 speech perception and, hence, also production, especially in the case of a strong similarity between sounds of the L1 and L2. Instead of a new L2 sound category coming into existence, L2 sounds may be mapped onto and represented by a phonetically similar L1 category. Research, such as eye-tracking studies, has shown, however, that the picture seems to be more complex than that. That is, an asymmetry between one’s lexical and perceptual representation has been emphasized, meaning that L2 speakers might have stored the contrast between items on the lexical level while still facing difficulties in perceiving the contrast (see Cutler et al., 2006).

Let us consider one specific example in this context, namely when native speakers of German are exposed to the English vowel contrast between /ɛ/ (DRESS vowel), as in *pen*, and /æ/ (TRAP vowel), as in *pan*. Remember that well-known varieties of English, such as Standard American or Southern British English, distinguish between the two front, unrounded vowels /ɛ/ and /æ/ (see Altendorf and Watt, 2008; Kretzschmar, 2008). The two vowels are distinct in (at least) two ways. First, /ɛ/ is located slightly higher and more anterior in the vowel space, and therefore shows a lower F1 and a higher F2 value than /æ/. Second, /ɛ/ is shorter than /æ/ (see Bohn and Flege, 1990, 1992; Reetz and Jongman, 2009).^1^

Since /æ/ is not part of the Standard German vowel inventory, it frequently poses a challenge to learners of English, who may not distinguish between English /ɛ/ and /æ/ and/or rely on a close category they are familiar with from German, such as /ɛ/ or /ɛː/ for *pen* and *pan*, respectively (see Bohn and Flege, 1990, 1992; Flege, 1995; Kautzsch, 2010; Llompart and Reinisch, 2017, 2019; Hickey, 2019).^2^ Note that Dutch does not have /æ/ either and that several contributions consider how L1 Dutch speakers deal with this particular vowel challenge in English (e.g., Weber and Cutler, 2004; Escudero et al., 2008; Broersma and Cutler, 2011; Simon and D’Hulster, 2012). Weber and Cutler (2004) suggest that the distinction between L2 English /ɛ/-/æ/ is indeed represented at the lexical level although listeners cannot successfully differentiate between the two vowels in perception. In eye-tracking experiments, the authors found that Dutch L1 but not English L1 listeners, who saw the images of a panda and a pencil and had been asked to click on the picture of the panda, tended to look at the image of the pencil during the presentation of the first syllable of *panda*. When the task was to click on the image of the pencil, Dutch L1 listeners behaved similarly to English L1 listeners, that is, the picture of the panda did not compete with that of the pencil in the same way. For our purpose, it is important to observe that /æ/, as well as the contrast between /ɛ/ and /æ/, which are not found in German (or Dutch), pose a possible challenge to L1 German (or Dutch) speakers. The objective of the present paper is to expand previous research by investigating whether some L1 German speakers show fewer difficulties in identifying /æ/ and discriminating /æ/ and /ɛ/ than others. That is, we examine whether the properties of one’s L1 vowel inventory affect the way one deals with the two English vowels /ɛ/ and /æ/. More precisely, we ask whether the presence/absence of the distinction between the two German vowels /eː/-/ɛː/ in a person’s L1 vowel space facilitates the identification of /æ/ and the discrimination of the English contrast between /ɛ/ and /æ/. We do so by examining both binary (identification rate of English /æ/ in the identification and reaction accuracy in the discrimination task) and continuous data (reaction times) in order to get a comprehensive picture and detect potential burdens in the processing of L2 sounds. Before going into detail, however, we consider, more generally, which factors might affect how a person acquires speech in an L2.

### 1.1. Relevant factors in the acquisition of speech in an L2

In this section, we discuss several factors that can affect the acquisition of speech in an L2. The factors are age and amount of experience (section 1.1.1) and similarities between a person’s L1 and the L2 (section 1.1.2).

#### 1.1.1. Age and amount of experience

A first factor is the age at which a person acquires the L2. If the acquisition of the L2 starts later, it is often more difficult to hide a foreign accent, that is, the L1 shapes L2 speech more than if the L2 acquisition begins at an earlier age (see Flege, 1992, 1995; Munro et al., 1996; for discussion, see also Birdsong, 2006). For instance, German-speaking children (L1) are more sensitive to the differences between the English /æ/ and a near German vowel (/ɛ/) than German-speaking adults (L1; see Weiher, 1975; Butcher, 1976 both cited in Flege, 1995).

A second factor is the amount of experience a person has in the L2. Usually, individuals with more experience in the L2 sound more native-like (see Best and Strange, 1992; Flege, 1995; Baker and Trofimovich, 2005). For example, Bohn and Flege (1992) show that German speakers of English who had spent on average about 7 to 8 months in an English-speaking environment barely distinguished between /ɛ/ and /æ/ in English, while German speakers of English who had spent on average 7.5 years in an English-speaking environment clearly separated between the two vowels (for related results, see Kautzsch, 2010; Simon and D’Hulster, 2012). The German speakers with longer exposure to English were more similar to English native speakers in that they produced a greater height difference between /ɛ/ and /æ/ in English than the German speakers with less experience in English. Also, the /æ/ of the German speakers with a low amount of experience in English was articulated higher than the /æ/ of German speakers with more experience in English and English native speakers.

Although the two factors age and amount of experience play a crucial role in the acquisition of an L2 in general and with regard to how much the L1 shapes the L2, we see that even highly advanced language users still differ from native speakers. For instance, Bohn and Flege (1992) show that even the German group with a long experience in L2 English differs in some ways from the group of English native speakers (for more discussion, see also Hickey, 2019). That is, first, in terms of spectral properties, the English /ɛ/ produced by the German speakers with a lot of experience in English was not different from their German /ɛ/ and /ɛː/, although at least some research suggests subtle spectral differences between the English and the German version of the vowel (see Bohn and Flege, 1990). Second, the German speakers with much experience in English differed from the English native speakers with regard to vowel duration, too. Both German groups, the inexperienced and the experienced one, pronounced English /æ/ clearly shorter than native speakers of English, and the German speakers with a high level of experience in English realized English /ɛ/ shorter than the English native speakers. These results illustrate the complexity of the issue: on the one hand, the durations of the two English vowels were not native-like even in the German group with a high level of experience in English; on the other hand, this group expressed the expected *duration difference* between the two English vowels and was, so to speak, native-like with respect to the *duration contrast*. A further finding from Bohn and Flege (1992) indicated that higher experience in an L2 does not necessarily lead to sounding more native-like. In an intelligibility test, they found that both words with /ɛ/ and words with /æ/ were less intelligible if pronounced by any of the two German groups (inexperienced or experienced in English) in comparison to the group of English native speakers (see also Flege et al., 1997).

#### 1.1.2. Similarities between a person’s L1 and the L2

A third factor is the degree of similarity between specific phenomena in the L1 and L2. It has been stated that a higher degree of similarity between phenomena in the L1 and L2 typically results in greater L1 influence on the L2 and more difficulties in correctly acquiring the L2 sound (see Flege, 1987, 1995; for discussion, see also Baker and Trofimovich, 2005). Bohn and Flege (1990) keep the adjective “similar” apart from “new” and “identical”. For instance, they consider /m/ to be identical in German and English, /u/ to be similar, and /æ/ to be a new sound for German learners of English. It goes without saying, however, that even a new L2 sound, as English /æ/, can be articulatorily and acoustically close to an L1 category. Since both English /ɛ/ and /æ/ as well as German /ɛ/ and /ɛː/ are front, open-mid, and unrounded vowels, they are acoustically and articulatorily quite similar. In an L2, individuals might fail to detect these fine acoustic differences between two sounds of the L2 or between sounds in the L1 and those in the L2, which can cause the assimilation of actually distinct sounds into a single category in the L2 (see Flege, 1995; but see also discussion above). Hence, the creation of a category for the L2 sound arises only if the respective differences and fine details are processed, which is, in turn, more likely if L1 and L2 sounds differ more clearly (see Flege, 1995).

#### 1.1.3. Intermediate summary

To reach an intermediate summary, we can say that whether and to what extent the L1 affects the acquisition of the L2 has been widely examined in the literature, and several factors have been taken into consideration. However, we feel that another factor has been mostly neglected, namely the role of sociolinguistic aspects of an individual’s L1 in the context of L2 speech acquisition. It is the aim of the present paper to investigate this issue. Precisely, we aim at investigating whether the degree to which German native speakers distinguish between the two front, unrounded vowels /eː/ (e.g., *dehnen*, [ˈdeːnən], “to stretch”) and /ɛː/ (e.g., *Dänen*, [ˈdɛːnən], “the Danish”) in their L1 has an impact on how well these individuals identify /æ/ and discriminate between the two English front, unrounded vowels /ɛ/ (e.g., *pen*, [pɛn]) and /æ/ (e.g., *pan*, [pæn]). We therefore intend to expand previous research on how a high degree of similarity between a phenomenon in the L1 and one in the L2 affects L2 speech acquisition in detail, by considering sociophonetic variation across different native speakers of German.

### 1.2. The /eː/-/ɛː/ contrast in (standard) German

Let us now consider in more detail the vowel contrast between /eː/ and /ɛː/ in German, which we hypothesize to affect how learners of English deal with the two English vowels /ɛ/ and /æ/. In Standard German, the two front, unrounded vowels /eː/ (close-mid vowel as in *dehnen*, [ˈdeːnən], “to stretch”) and /ɛː/ (open-mid vowel as in *Dänen*, [ˈdɛːnən], “the Danish”) are distinct phonemes (see Wiese, 2000; Fuhrhop and Peters, 2023). However, there is empirical evidence suggesting that some speakers do not differentiate between the two vowels. This tendency toward a merger of the two vowels depends on several factors. The most important factor is the regional origin of the speaker: we know that /ɛː/ is (largely) replaced by /eː/ in northern Germany (e.g., Ramers, 1988; Kohler, 1995; Ternes, 1999; for empirical studies, see König, 1989; Kleiner, 2011; Elmentaler and Rosenberg, 2015). In one study, only minor acoustic differences between the two vowels were found, which can be attributed to the fact that most speakers investigated in this study were from northern Germany (Simpson, 1998). Most other acoustic analyses show differences in the pronunciation of the two vowels for speakers from different regions in Germany (Sendlmeier and Seebode, 2006; Schoormann et al., 2019; Predeck et al., 2021). Results from the acoustic analysis of the corpus *German Today* (Kleiner, 2011, 2015) exhibit the tendency toward a merger in northern and eastern Germany as well as in Austria, while the south-west of Germany and Switzerland maintain a distinction (see Frank, in preparation). Critically, we find variation in each region, thus no region exhibits a complete merger in production.

The second factor is the degree of formality in a speech situation, that is, the vowels are distinguished more clearly in formal speech (Stearns and Voge, 1979; König, 1989; Kleiner, 2011). Elmentaler and Rosenberg (2015), however, found no significant differences between reading pronunciation, interviews, and informal conversations among family and friends in northern Germany. The third factor is the phonological context. The opposition between the two vowels before /ʁ/ is usually neutralized (Stearns and Voge, 1979; Wiese, 2000) and there is evidence for a merger both toward /εː/ (Wiese, 2000) and toward /eː/ (Heffner, 1965; Kohler, 1995). While the work taken into account so far concentrates on production, some perception experiments exist, too. A few studies deal with the salience of the vowel merger toward /eː/. These studies show that the vowel merger is classified by listeners as Standard German and not as a noticeable deviation, that is, listeners perceive the pronunciation of /ɛː/ as [eː] as Standard German (Elmentaler and Rosenberg, 2015; Hettler, 2017; Kiesewalter, 2019). In another perception experiment (Block et al., 2023), listeners from different regions could reliably identify vowel stimuli from speakers without a merger, whereas identification was more difficult for stimuli from speakers with a merger (for a further study on the perception of the two vowels, see Frank, in preparation).

### 1.3. Objectives and hypotheses

In sum, while English has both /ɛ/ and /æ/, German does not have /æ/ but both /ɛ/ and /ɛː/. German /ɛ/ might be a candidate to express English /ɛ/ in the L2, due to the acoustic similarities of the two. German /ɛː/, in turn, could be a substitute for English /æ/, since German /ɛː/ is longer than German /ɛ/ and since English /æ/ is longer than English /ɛ/. A remaining question is then, however, what happens if L1 German speakers merge /ɛː/ with /eː/. In this case, L1 German speakers have /ɛ/, but not /ɛː/, in their vowel inventory. Here, we aim at expanding previous research on how the L1 affects the acquisition of L2 speech by specifically focusing on the role of sociophonetic variation in the L1. Native speakers of German vary with respect to the degree of overlap between the two vowels /eː/ and /ɛː/: there are speakers who maintain the contrast (henceforth: /eː/-/ɛː/ maintainers) and speakers who merge the two vowels (henceforth: /eː/-/ɛː/ mergers). We ask whether these speaker groups identify /æ/ and discriminate the English vowels /ɛ/ and /æ/ differently in speech perception. Assuming that there are two groups, /eː/-/ɛː/ maintainers and /eː/-/ɛː/ mergers, we test the following hypotheses.

The identification of English /æ/ and the discrimination of the English /ɛ/−/æ/ contrast is:

H0: The same in both groups.

H1: More native-like in the group of /eː/-/ɛː/ maintainers.

These hypotheses derive from the patterns summarized in Table 1. Table 1 shows that we expect both /eː/-/ɛː/ maintainers and /eː/-/ɛː/ mergers to assimilate English /ɛ/ to German /ɛ/ since both groups are equipped with /ɛ/ in their native (German) vowel inventory. The situation is different for English /æ/. On the one hand, the groups could form the new category /æ/; on the other hand, they could rely on a close vowel they are familiar with from their native vowel inventory. While for /eː/-/ɛː/ maintainers, the most likely candidate is /ɛː/, for /eː/-/ɛː/ mergers, the most likely candidate is /ɛ/. If /eː/-/ɛː/ maintainers and /eː/-/ɛː/ mergers have developed the new category /æ/, we should not expect different identification / discrimination patterns for the two speaker groups (H0), they should perform well in the identification of /æ/ / discrimination between /ɛ/ and /æ/. If, however, /eː/-/ɛː/ maintainers assimilate English /æ/ to German /ɛː/ and /eː/-/ɛː/ mergers assimilate English /æ/ to German /ɛ/, we should expect better identification / discrimination patterns for /eː/-/ɛː/ maintainers (H1). Although both /ɛ/ and /ɛː/ are distinct from /æ/ in terms of vowel quality, /ɛː/ resembles /æ/ more than /ɛ/ in terms of duration. Overall, the contrast between English /ɛ/-/æ/ would be neutralized in lexical entries only for /eː/-/ɛː/ mergers in this scenario (both of the two English vowels would be assimilated to German /ɛ/) but not for /eː/-/ɛː/ maintainers (who would assimilate English /ɛ/ to German /ɛ/ and English /æ/ to German /ɛː/). The neutralization of the contrast in /eː/-/ɛː/ mergers should prevent accurate identification / discrimination and the “better” performance of /eː/-/ɛː/ maintainers could be reflected in (a) more accurate and/or (b) faster identification / discrimination.

**Table 1:** German-speaking learners of English and the vowels /ɛ/ and /æ/.

English target vowel	/eː/-/ɛː/ maintainers	/eː/-/ɛː/ mergers
/ɛ/	Assimilation to /ɛ/	Assimilation to /ɛ/
/æ/	Assimilation to /ɛː/ or Development of /æ/	Assimilation to /ɛ/ or Development of /æ/

## 2. Methodology and results

### 2.1. Experiment I: identification task

In Experiment I, participants saw two pictures, heard a word, and had to decide which of the two concepts represented in the pictures they had heard.

#### 2.1.1. Participants

Fifty-one native speakers of German from northern Germany participated in the experiment [33 female, 18 male; mean age: 24.6 years (SD: 3.4 years; range: 19–35 years)].^3^ They had an academic background and English was their second language. They did not declare a speech disorder or hearing impairment, did not use a hearing aid, and could see clearly.

All participants were well-educated and had taken English as one of the central school subjects for at least 8 years. That is, all of them had a solid knowledge and command of English. Despite this similarity between our participants, they varied to some extent with regard to their level of English. We grouped our participants into three proficiency groups (“low,” “mid,” and “high” competence in English). Categorizing participants into the three groups was done in the following way. The first author of the paper and a native speaker of English listened to sound files of items, which were recorded in a short production experiment prior to the perception experiments. They examined the pronunciation in terms of nativelikeness, and assigned the participants to the groups “low,” “mid,” and “high.” If the first author and the native speaker of English did not agree, the case was discussed until agreement was reached. Our evaluation of the participants’ level of English is thus based on their pronunciation only. The participants’ level of English was later entered as a control variable into the statistical models.

A key element of our work is whether and to what extent our participants distinguish between the German vowels /eː/ and /ɛː/. In order to obtain an objective reflection of this, we relied on the data from the production experiment reported in Frank (in preparation). In that study, our 51 native speakers of German read a list of 174 words that included 19 items containing /eː/ (e.g., *dehnen*, [ˈdeːnən], “to stretch”) and 29 items containing /ɛː/ (e.g., *Dänen*, [ˈdɛːnən], “the Danish”) in Standard German. Frank (in preparation) analyzed F1 and F2, which, in turn, were then used to calculate a Pillai score (see Nycz and Hall-Lew, 2013). For each speaker, a Pillai score was calculated over all tokens. A score of “0” means that a speaker does not distinguish between the two vowels at all (= /eː/-/ɛː/ merger). A Pillai score of “1,” in turn, means that a person fully distinguishes the two vowels /eː/ and /ɛː/ (= /eː/-/ɛː/ maintainer). The German Pillai score entered our statistical models as a continuous fixed effect (see below). Remember from section 1.2 that, even though merging the two German vowels is quite common in northern Germany (where our participants are from), we still observe variation even within a region.^4^

#### 2.1.2. Materials

Two minimal pairs were selected for this experiment, namely *pan/pen* and *paddle/pedal*. The four words were spoken and recorded by a 24-year-old male US American from California in a silent room. The four sound files were normalized (70 dB) and subsequently used as the basis for the following manipulations. We created a spectral continuum with 11 steps from an extreme /æ/ (Step 1) to an extreme /ɛ/ (Step 11) based on a Praat script (Winn, 2019; Boersma and Weenink, 2021). Steps 3 and 9 represented the spectral information of the original /æ/ and /ɛ/, respectively. From Steps 3 and 9, we created two steps below (Steps 1 and 2 and Steps 7 and 8) and two steps above (Steps 4 and 5 and Steps 10 and 11). Step 6 was the middle of the continuum. The detailed spectral information is summarized in Table 2.

**Table 2:** Spectral information of the continuum.

Step	*pan/pen*	*paddle/pedal*
	(F1/F2 in Hz)	(F1/F2 in Hz)
1	724/1,706	839/1,531
2	708/1,715	811/1,542
3	692/1,725	783/1,553
4	676/1,734	755/1,564
5	660/1,744	728/1,576
6	645/1,753	702/1,587
7	629/1,763	676/1,599
8	614/1,773	651/1,610
9	599/1,782	626/1,622
10	585/1,792	602/1,633
11	570/1,802	578/1,645

In addition, three vowel duration categories, short, middle, and long, were used. We relied on the original duration of the /æ/ words to define the long duration and on the original duration of /ɛ/ to specify the short duration. The middle duration was then positioned between the two. The vowel durations are given in Table 3.

**Table 3:** Vowel durations (in ms) used in the experiment.

Vowel duration	*pan/pen*	*paddle/pedal*
Short	142	80
Middle	192	122
Long	242	164

#### 2.1.3. Procedure

The experiment was conducted in a silent room. Participants sat about 60 cm in front of a computer screen and wore KOSS R-80 over-ear headphones. We used E-Prime 3 (Psychology Software Tools, 2016) for stimulus presentation. In each trial, the participants saw two pictures on the screen, either the image of a pan and the image of a pen or the picture of a paddle and the picture of a pedal. Their task was to indicate (via button press on a regular keyboard) whether the word they had heard represented the first or second image. The position (left side vs. right side of the screen) of the pictures (pan vs. pen and paddle vs. pedal) was counterbalanced across participants. Participants were requested to press the left and right arrow on the keyboard to indicate that they believed they had heard the item on the left or right, respectively.

The two minimal pairs *pan/pen* and *paddle/pedal* were tested in separate blocks. Further, we distinguished between three blocks for each minimal pair, one for each vowel duration type (short, middle, long). Hence, the identification task consisted of six distinct blocks (2 minimal pairs × 3 vowel duration types per minimal pair). The three blocks relating to one minimal pair always appeared one after the other in the same order (middle, short, long). All the *pan/pen* blocks appeared first, all the *paddle/pedal* blocks afterwards. Each person was tested on 198 trials (2 minimal pairs × 3 vowel duration types × 11 steps of the continuum × 3 times each sound file). Hence, each of the six blocks contained 33 cases (11 steps of the continuum × 3 times each sound file).

#### 2.1.4. Data analysis

The two response variables were the percentage of the selection of the picture representing the /æ/ word (Selection of /æ/) and ReactionTime. We used a binomial logistic regression to examine the first and linear mixed effects models to analyze the second one in R (R Core Team, 2021), using the lme4 (Bates et al., 2015) and lmerTest packages (Kuznetsova et al., 2017).^5^ In the analysis of ReactionTime, we first log transformed (to the base 10) the values (see Winter, 2020). We then checked the dataset for extreme values (statistical outliers), defined as the overall mean plus/minus 2.5 standard deviations (see Loewen and Plonsky, 2016). No extreme values were detected.

Model fitting proceeded in the following way (fit with ML or REML; see Field et al., 2012). On the level of random effects, we started with the structure containing the intercepts for Subject and Item as well as the slope of our central fixed effect, GermanPillai, by Item (see Winter, 2020). Complex random effects structures can lead to a statistically unstable analysis, that is, convergence issues can arise (see Barr et al., 2013; Matuschek et al., 2017; Cohen and Kang, 2018). If this was the case, we first changed the optimizer to “bobyqa” (see Winter, 2020). If this was not enough, this initial structure was reduced manually and checked again, first without and, if necessary, with the different optimizer (bobyqa). The initial model was reduced step by step until convergence was reached.

On the level of the fixed effects structure, we included the German Pillai score (GermanPillai), Step (1–11, factor-transformed, see Winter, 2020), Duration (short, middle, long), LevelOfEnglish (low, mid, high), and the three interactions GermanPillai × Step, GermanPillai × Duration, and GermanPillai × LevelOfEnglish in the initial model. If a fixed effect was not significant, it was removed from the model. The new model, without the excluded fixed effect, was then checked again. If significance was detected, the respective case was further investigated using three criteria mentioned in Plag et al. (2017). Only if all of the three criteria went in the expected direction, we considered the case to be significant and retained the fixed effect in the model. The first criterion implied that the t statistics of the respective fixed effect had to be lower than −2 or higher than 2.^6^ Second, the model with the fixed effect had to have a better fit than the model without it; this would be reflected in a significant difference between the two models, as detected in an ANOVA. Third, the Akaike Information Criterion (AIC) had to be smaller for the model with, in contrast to the model without, the respective fixed effect (see also Pinheiro and Bates, 2000; Wu, 2010).

Finally, for the categorical fixed effects, planned pairwise comparisons (Tukey tests) were conducted using the emmeans package (Lenth, 2020) in R to shed light on the comparisons that we cannot see in the model, that is, in Tables 4, 5. This method has been used in the context of different kinds of regression analyses, such as logistic / ordinal regression (see Montrul et al., 2019; Kim and Yoon, 2020).

**Table 4:** Fixed effects statistics of selection of /æ/.

	Estimate	Std. error	z value	Pr(>|z|)
(Intercept)	0.37	0.30	1.24	>0.05
GermanPillai	2.80	0.46	6.12	<0.001^***^
Duration_middle	−0.01	0.16	−0.05	>0.05
Duration_short	−0.28	0.16	−1.79	>0.05
Step_2	0.30	0.32	0.96	>0.05
Step_3	0.47	0.31	1.50	>0.05
Step_4	0.43	0.31	1.39	>0.05
Step_5	0.20	0.30	0.66	>0.05
Step_6	0.20	0.30	0.68	>0.05
Step_7	−0.17	0.30	−0.58	>0.05
Step_8	−0.14	0.30	−0.47	>0.05
Step_9	−0.09	0.31	−0.31	>0.05
Step_10	−0.79	0.31	−2.52	<0.05^*^
Step_11	−1.03	0.32	−3.20	<0.01^**^
GermanPillai × Duration_middle	−0.18	0.25	−0.72	>0.05
GermanPillai × Duration_short	−0.84	0.25	−3.39	<0.001^***^
GermanPillai × Step_2	−0.95	0.52	−1.83	>0.05
GermanPillai × Step_3	−1.59	0.51	−3.12	<0.01^**^
GermanPillai × Step_4	−1.98	0.50	−3.97	<0.001^***^
GermanPillai × Step_5	−2.41	0.49	−4.94	<0.001^***^
GermanPillai × Step_6	−3.03	0.49	−6.24	<0.001^***^
GermanPillai × Step_7	−3.14	0.49	−6.44	<0.001^***^
GermanPillai × Step_8	−3.70	0.49	−7.51	<0.001^***^
GermanPillai × Step_9	−4.25	0.50	−8.49	<0.001^***^
GermanPillai × Step_10	−3.53	0.51	−6.89	<0.001^***^
GermanPillai × Step_11	−3.55	0.53	−6.73	<0.001^***^

*p* levels: * <0.05, ** <0.01, *** <0.001.

**Table 5:** Fixed effects statistics of ReactionTime.

	Estimate	Std. error	df	t value	Pr(>|t|)
(Intercept)	3.09e + 00	4.41e − 02	2.43e + 01	70.09	<0.001^***^
GermanPillai	−1.18e − 01	6.07e − 02	5.28e + 01	−1.94	>0.05
Duration_middle	9.78e − 02	1.32e − 02	1.00e + 04	7.44	<0.001^***^
Duration_short	−2.03e − 02	1.32e-02	1.00e + 04	−1.55	>0.05
Step_2	−1.57e − 02	8.50e − 03	1.00e + 04	−1.85	>0.05
Step_3	−2.72e − 03	8.50e − 03	1.00e + 04	−0.32	>0.05
Step_4	2.03e − 03	8.50e − 03	1.00e + 04	0.24	>0.05
Step_5	7.06e − 03	8.50e − 03	1.00e + 04	0.83	>0.05
Step_6	2.03e − 02	8.50e − 03	1.00e + 04	2.39	<0.05^*^
Step_7	1.24e − 02	8.50e − 03	1.00e + 04	1.45	>0.05
Step_8	1.46e − 02	8.50e − 03	1.00e + 04	1.72	>0.05
Step_9	9.15e − 03	8.50e − 03	1.00e + 04	1.08	>0.05
Step_10	3.21e − 03	8.50e − 03	1.00e + 04	0.38	>0.05
Step_11	−3.56e − 03	8.50e − 03	1.00e + 04	−0.42	>0.05
GermanPillai × Duration_middle	−4.05e − 02	2.01e − 02	1.00e + 04	−2.02	<0.05^*^
GermanPillai × Duration_short	8.43e − 03	2.01e − 02	1.00e + 04	0.42	>0.05

*p* levels: * <0.05, *** <0.001.

#### 2.1.5. Results

**Figure 1:**
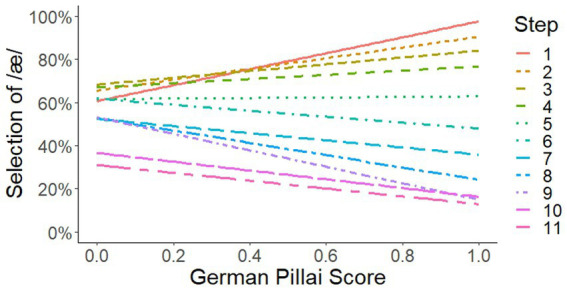
Interaction of GermanPillai × Step.

**Figure 2:**
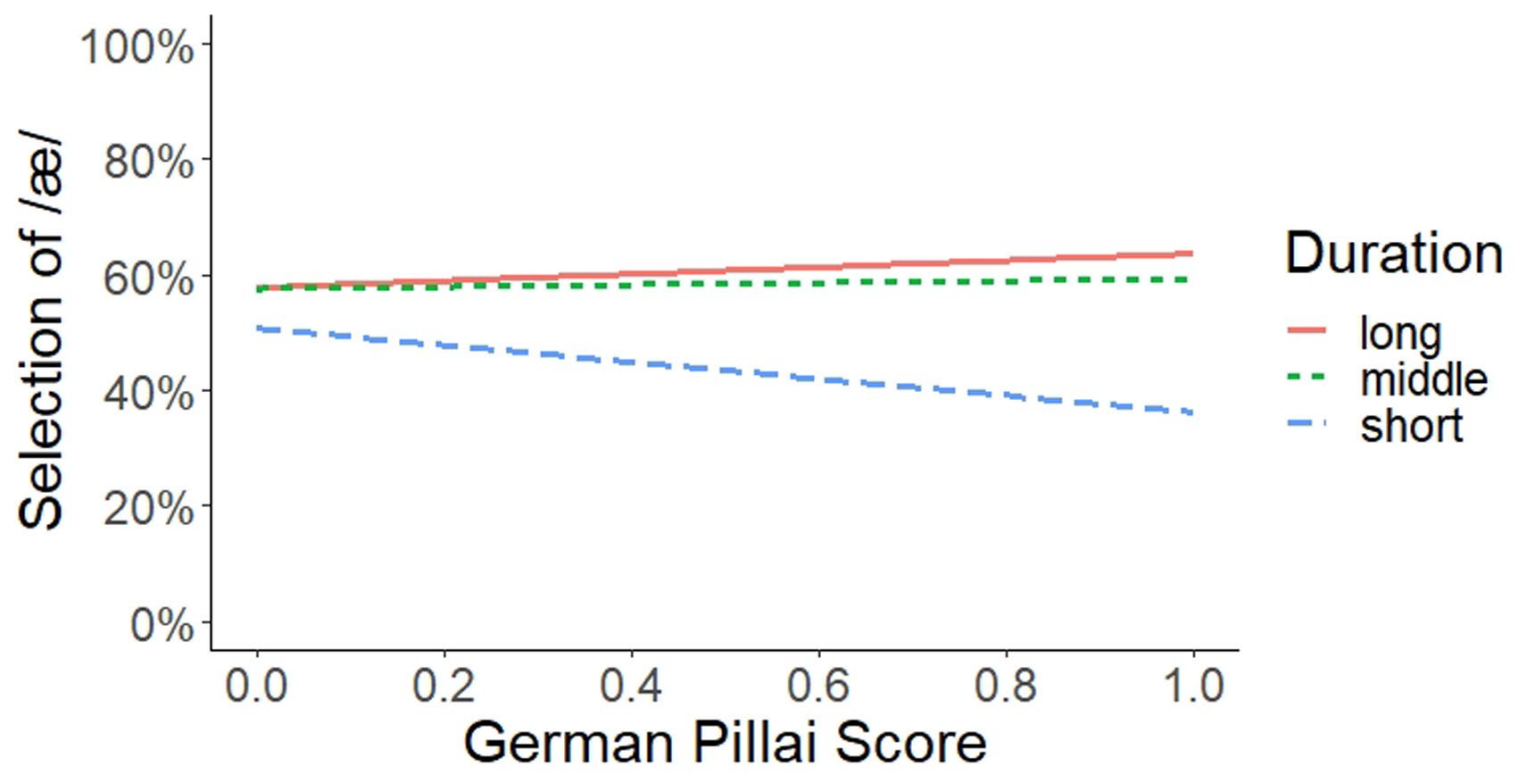
Interaction of GermanPillai × Duration.

We start presenting the results for Selection of /æ/. The most important results are the two significant interactions GermanPillai × Step and GermanPillai × Duration (see Figures 1, 2). The statistical details are presented in Tables 4, 6.

**Table 6:** Random effects statistics of selection of /æ/.

	Variance	Standard deviation
Subject (intercept)	0.11	0.34
Item (intercept)	0.02	0.15

We see two significant interactions, GermanPillai × Step and GermanPillai × Duration. Let us start with the first one. In Figure 1, we see that the vertical sequence of the profiles matches the sequence of steps, hence the materials and manipulations worked out as intended. On the horizontal level, we observe that the manipulations of the acoustic signal had a considerably smaller effect on the response behavior of participants at the left than at the right margin. At the left margin, where we find /eː/-/ɛː/ mergers, differences in vowel quality bring about a change in identification of /æ/ between roughly 30% and 60%–70%. That is, /eː/-/ɛː/ mergers identified extreme renditions of /æ/ as /æ/ in only 60–70%. Likewise, /eː/-/ɛː/ mergers identified extreme renditions of /ɛ/ as /ɛ/ in only 70%. At the right margin, where we find /eː/-/ɛː/ maintainers, differences in vowel quality bring about a change in identification of /æ/ between roughly 20% and 90%. That is, /eː/-/ɛː/ maintainers were much more sensitive to the manipulations, they identified extreme renditions of /æ/ as /æ/ in about 90%. Likewise, /eː/-/ɛː/ maintainers identified extreme renditions of /ɛ/ as /ɛ/ in about 80%.

In Figure 2, the second interaction is plotted. We see again that the vertical sequence of the profiles matches the sequence of the three duration types (from long to middle to short), indicating that the materials and manipulations worked out as intended. On the horizontal level, we observe again that the manipulations of the signal had a considerably smaller effect on the response behavior of participants at the left than at the right margin. At the left margin, differences in duration bring about a change in identification of /æ/ between roughly 55% and 60%. That is, /eː/-/ɛː/ mergers identified long stimuli as /æ/ in about 60%. Short stimuli, in turn, were identified as /ɛ/ in about 45%. At the right margin, however, differences in duration bring about a change in identification of /æ/ between roughly 40 and 60%. Long stimuli were identified as /æ/ in about 60% while short stimuli were identified as /ɛ/ in about 60%. Overall, while for the /eː/-/ɛː/ mergers the percentage of /æ/ selection was rather similar for short and long vowels, /eː/-/ɛː/ maintainers clearly selected /æ/ more often for long than for short vowels.

**Figure 3:**
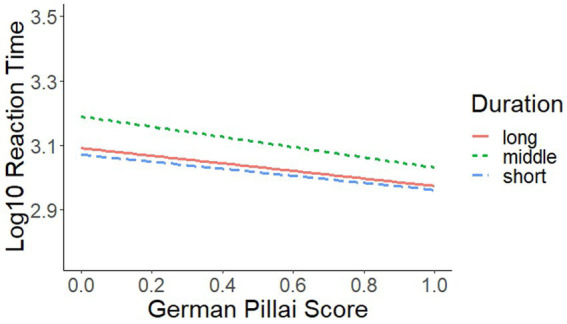
Interaction of GermanPillai × Duration.

We now turn to the analysis of ReactionTime, where we found a significant interaction of GermanPillai × Duration (see Figure 3; Tables 5, 7).

**Table 7:** Random effects statistics of ReactionTime.

	Variance	Standard deviation
Subject (intercept)	0.01	0.09
Item (intercept)	0.00	0.03
Residual	0.03	0.18

In the analysis of ReactionTime, there is a main effect of Step. Participants reacted more quickly to Step 1 than to Step 6. A Tukey *post hoc* test revealed further that participants responded faster to Step 2 than to Step 6 (estimate = −0.04, SE = 0.01, df = Inf, z ratio = −4.24, *p* < 0.01), to Step 2 than to Step 7 (estimate = −0.03, SE = 0.01, df = Inf, z ratio = −3.30, *p* < 0.05), and to Step 2 than to Step 8 (estimate = −0.03, SE = 0.01, df = Inf, z ratio = −3.57, *p* < 0.05).

Furthermore, we also detected a significant interaction, namely GermanPillai × Duration. In Figure 3, we see, first, that /eː/-/ɛː/ maintainers (at the right margin) were generally quicker than /eː/-/ɛː/ mergers (at the left margin). Second, in comparison to long and short stimuli, /eː/-/ɛː/ mergers needed more time to deal with the intermediate category. Although /eː/-/ɛː/ maintainers needed more time to react to the intermediate category, too, the difference between the intermediate category and the long and short stimuli was smaller for this group of speakers.

### 2.2. Experiment II: discrimination task

In Experiment II, participants heard two sound files and were asked to indicate whether the two were acoustically identical or different.

#### 2.2.1. Participants

The participants were those from Experiment I.

#### 2.2.2. Materials

The materials/sound files were those from Experiment I.

#### 2.2.3. Procedure

The experiment was conducted in a silent room. Participants sat about 60 cm in front of a computer screen and wore KOSS R-80 over-ear headphones. We used E-Prime 3 for stimulus presentation. In each trial, participants heard two sound files and indicated via button press, using the left and right arrow on a regular keyboard, whether they believed they were identical or different. We counterbalanced the association of arrow and identical/different across participants. As in Experiment I, both the two minimal pairs and the three vowel duration types appeared in different blocks. The spectral distance between the two sound files of a trial was 3, 2, 1, or 0 steps. That is, for instance, if the first sound file contained Step 3 and the second sound file Step 4, the distance between the two was 1 step. The interstimulus interval (ISI) was 1 s.^7^ Each person was tested on 390 trials (2 minimal pairs × 3 vowel duration types × 65 trials per block).

#### 2.2.4. Data analysis

The two response variables were Accuracy and ReactionTime. We used binomial logistic regression to examine the first and linear mixed effects models to analyze the second one in R, using the lme4 and lmerTest packages. In the analysis of ReactionTime, only correct answers were considered (40% of the answers), following common practice (see Jiang, 2012, p. 68–69). The reaction time data was log transformed (to the base 10), before we additionally checked it for extreme values. No extreme value was detected (see also section 2.1.4).

On the level of the random effects structure, we proceeded in the way described in section 2.1.4. On the level of the fixed effects structure, we included GermanPillai, StepDistance (0, 1, 2, 3; factor-transformed), Duration (short, middle, long), LevelOfEnglish (low, mid, high), and the three interactions GermanPillai × StepDistance, GermanPillai × Duration, and GermanPillai × LevelOfEnglish in the initial model. The model was reduced step by step and significances were checked using the technique outlined in section 2.1.4. Planned pairwise comparisons were used as outlined in section 2.1.4.

#### 2.2.5. Results

**Figure 4:**
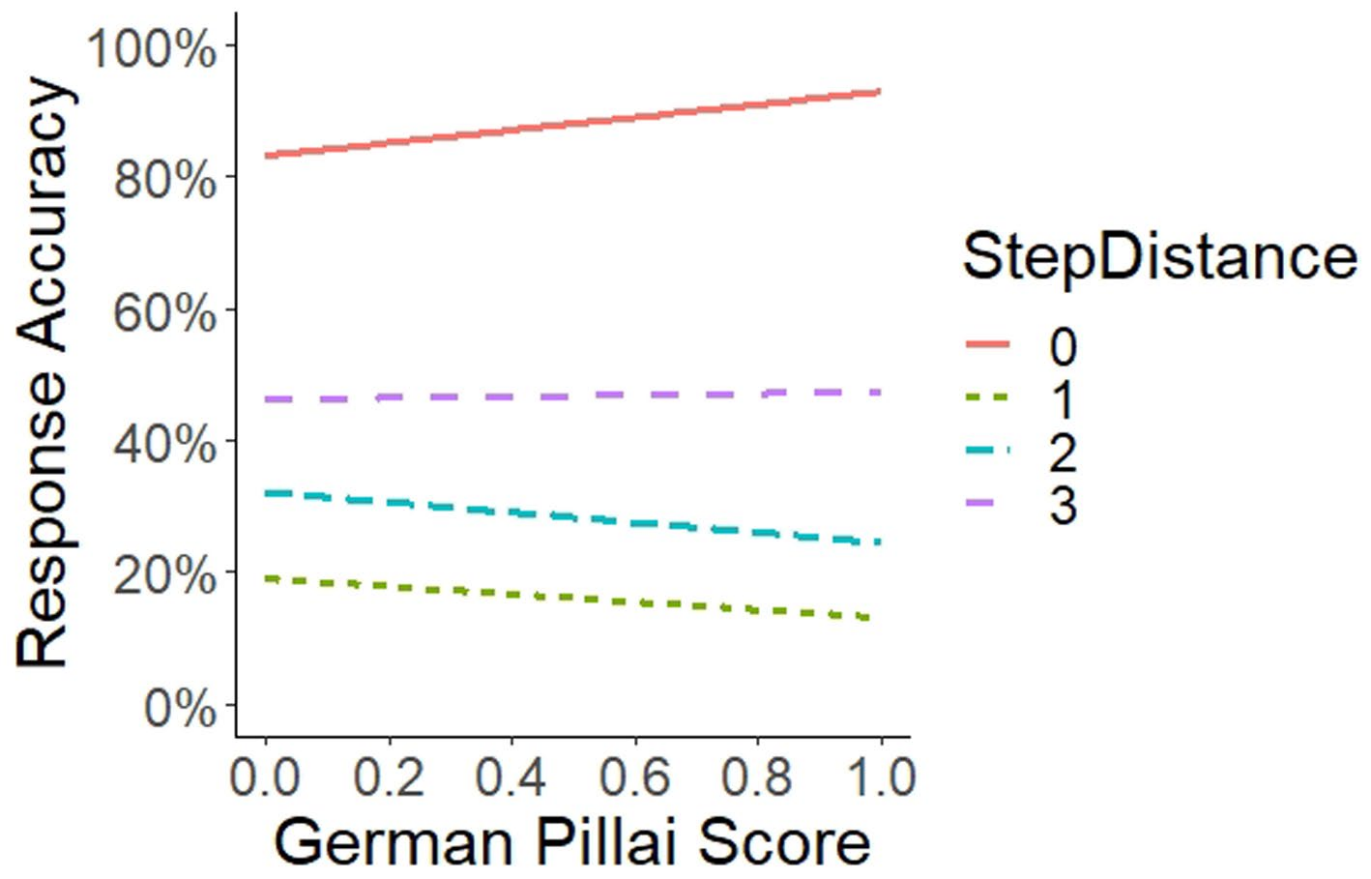
Interaction of GermanPillai × StepDistance.

We start looking at accuracy. Here, the interaction GermanPillai × StepDistance reached significance (see Figure 4). The statistical details are presented in Tables 8, 9.

**Table 8:** Random effects statistics of accuracy.

	Variance	Standard deviation
Subject (intercept)	0.36	0.60
Item (intercept)	0.01	0.08

**Table 9:** Fixed effects statistics of accuracy.

	Estimate	Std. error	z value	Pr(>|z|)
(Intercept)	1.63	0.29	5.58	<0.001^***^
Duration_middle	−0.01	0.04	−0.30	>0.05
Duration_short	−0.18	0.04	−4.12	<0.001^***^
GermanPillai	0.94	0.44	2.14	<0.05^*^
StepDistance_1	−3.01	0.18	−16.85	<0.001^***^
StepDistance_2	−2.31	0.17	−13.47	<0.001^***^
StepDistance_3	−1.71	0.17	−10.10	<0.001^***^
GermanPillai * StepDistance1	−1.38	0.28	−4.89	<0.001^***^
GermanPillai * StepDistance2	−1.32	0.27	−4.88	<0.001^***^
GermanPillai * StepDistance3	−0.90	0.27	−3.37	<0.001^***^

*p* levels: * <0.05, *** <0.001.

In the analysis of Accuracy, we found a main effect of Duration, indicating that short vowels were responded to less accurately than long ones. A Tukey *post hoc* test further showed that middle vowels were responded to more accurately than short vowels (estimate = 0.16, SE = 0.04, df = Inf, z ratio = 3.82, *p* < 0.001).

Further, we detected a significant interaction GermanPillai × StepDistance. We see in Figure 4 that, if the StepDistance was 0, that is, if the sound files a person was exposed to in a trial were identical, and in contrast to if the StepDistance was 1, 2, or 3, /eː/-/ɛː/ maintainers (at the right margin) responded slightly more accurately than /eː/-/ɛː/ mergers. For StepDistances of 1 and 2, however, /eː/-/ɛː/ mergers (at the left margin) reacted slightly more accurately than /eː/-/ɛː/ maintainers.

We now turn to ReactionTime (see Tables 10, 11). The analysis of ReactionTime revealed two main effects, one of Duration and one of StepDistance, but no significant interaction. First, the response time was longer when the vowel had a middle duration in comparison to when it had a long duration. Also, reaction times were shorter for short than for long vowels. A Tukey *post hoc* test indicated that the response time was longer for middle than for short vowel durations (estimate = 0.03, SE = 0.00, df = Inf, z ratio = 11.98, *p* < 0.001). Second, participants needed more time to respond when the StepDistance between the two sound files of a trial was 1, 2, or 3, in comparison to when it was 0. A Tukey *post hoc* test showed that the response times were longer for a StepDistance of 1, compared to StepDistances of 2 and 3 (1 vs. 2: estimate = 0.01, SE = 0.00, df = Inf, z ratio = 2.94, *p* < 0.05; 1 vs. 3: estimate = 0.03, SE = 0.00, df = Inf, z ratio = 8.28, *p* < 0.001). Also, participants responded more slowly for StepDistance 2 than for StepDistance 3 (estimate = 0.02, SE = 0.00, df = Inf, z ratio = 5.95, *p* < 0.001).

**Table 10:** Random effects statistics of ReactionTime.

	Variance	Standard deviation
Subject (intercept)	0.00	0.04
Item (intercept)	0.00	0.02
Residual	0.01	0.08

**Table 11:** Fixed effects statistics of ReactionTime.

	Estimate	Std. error	df	t value	Pr(>|t|)
(Intercept)	3.42e + 00	1.29e − 02	1.62e + 00	264.57	<0.001^***^
Duration_middle	1.22e − 02	2.21e − 03	7.85e + 03	5.50	<0.001^***^
Duration_short	−1.48e − 02	2.25e − 03	7.85e + 03	−6.60	<0.001^***^
StepDistance_1	3.57e − 02	2.99e − 03	7.87e + 03	11.92	<0.001^***^
StepDistance_2	2.61e − 02	2.58e − 03	7.87e + 03	10.13	<0.001^***^
StepDistance_3	1.04e − 02	2.29e − 03	7.87e + 03	4.53	<0.001^***^

*p* levels: *** <0.001.

## 3. Discussion

The present paper reported on an identification and a discrimination task and aimed at investigating whether the degree of overlap between the vowels /eː/ and /ɛː/ in L1 German affects the way individuals identify the new vowel /æ/ and discriminate between the vowels /ɛ/ and /æ/ in L2 English. We distinguished broadly between /eː/-/ɛː/ maintainers, that is, L1 German speakers who articulate the vowel contrast in their L1, and /eː/-/ɛː/ mergers, that is, L1 German speakers who do not articulate the vowel contrast in their L1.

In the identification task, we found that Step 1 was reacted to faster than Step 6 and Step 2 was responded to more quickly than Steps 6, 7, and 8. That is, some of the more extreme steps were easier to process. The significant interaction GermanPillai × Duration for the reaction time data signaled that /eː/-/ɛː/ mergers needed more time to process the difficult intermediate category (middle duration), in comparison to the categories of short and long duration, than /eː/-/ɛː/ maintainers.

Moreover, and crucially, we detected two significant interactions, namely GermanPillai × Duration and GermanPillai × Step in the data for the Selection of /æ/. Considering the first interaction, we saw that while /eː/-/ɛː/ maintainers selected English /æ/ slightly more often than /eː/-/ɛː/ mergers for long vowels, /eː/-/ɛː/ mergers picked English /æ/ slightly more frequently than /eː/-/ɛː/ maintainers for short vowels. Importantly, while /eː/-/ɛː/ mergers chose /æ/ for short and long vowels roughly equally often, /eː/-/ɛː/ maintainers selected this vowel more frequently for long than for short vowel durations. This supports H1: /eː/-/ɛː/ maintainers are more native-like in the identification of the new vowel /æ/ than /eː/-/ɛː/ mergers. While both speaker groups assimilate English /ɛ/ to German /ɛ/, the two groups seem to differ with regard to how they treat English /æ/. /eː/-/ɛː/ mergers, who only rely on /ɛ/ in L1 German and do not distinguish between /ɛ/ and /ɛː/, seem to assimilate English /æ/ to German /ɛ/, they fail to make adequate use of the duration parameter. /eː/-/ɛː/ maintainers perform more successfully for the parameter of vowel duration, which might derive from the fact that they are familiar with the distinction between /ɛ/ and /ɛː/ from the L1 German. /eː/-/ɛː/ maintainers either could have assimilated English /æ/ to German /ɛː/ or could have developed the new category /æ/.^8^ We will come back to this shortly.

Interestingly, the second interaction supports H1, too, although /eː/-/ɛː/ maintainers should not necessarily have an advantage when it comes to vowel quality (since both /eː/-/ɛː/ maintainers and /eː/-/ɛː/ mergers do have L1 German /ɛ/, but neither group has /æ/). We observed that /eː/-/ɛː/ maintainers selected English /æ/ more frequently than /eː/-/ɛː/ mergers for Steps 1 to 4. Step 5 shows a slight tendency for this, too. Acoustically speaking, these steps clearly represent English /æ/ and we have thus evidence that /eː/-/ɛː/ maintainers identify this vowel more accurately than /eː/-/ɛː/ mergers. This is further supported by the fact that /eː/-/ɛː/ mergers chose English /æ/ more frequently than /eː/-/ɛː/ maintainers for Steps 7 to 11, which carry the acoustic properties of English /ɛ/. Step 6, which is exactly in the middle of the continuum and can be considered to be neutral, joins for some reason the pattern we found for Steps 7 to 11. Overall, the significant interaction GermanPillai × Step we saw in the analysis of the response variable Selection of /æ/ in the identification task also supports H1: /eː/-/ɛː/ maintainers perform more native-like in the identification of /æ/ than /eː/-/ɛː/ mergers. Remember that we stated above that the results of the significant interaction GermanPillai × Duration could mean that /eː/-/ɛː/ maintainers either assimilated English /æ/ to German /ɛː/ or have developed the new category /æ/. The results of the second interaction, GermanPillai × Step, seem to be more compatible with the interpretation that /eː/-/ɛː/ maintainers have developed the new category /æ/. They outperform /eː/-/ɛː/ mergers not only on the level of vowel duration but also on the spectral level.

It is an interesting finding that vowel quality makes such a difference although both /eː/-/ɛː/ maintainers and /eː/-/ɛː/ mergers are familiar with the vowel quality of /ɛ/ but unfamiliar with the vowel quality of /æ/ from their L1 German. A potential explanation might be related to the fact that all of our participants were advanced speakers of English. Possibly, advanced speakers are generally aware of the differences between English /æ/ and German /ɛː/ / /ɛ/ (see also Weber and Cutler, 2004). Nevertheless, the two groups might differ with regard to what they still have to acquire. To become more native-like, /eː/-/ɛː/ maintainers must primarily tune their attention to vowel quality. For /eː/-/ɛː/ mergers, however, both vowel quality and duration are distinct from that of English /æ/. This might be an extra burden, a double burden so to speak, which /eː/-/ɛː/ mergers have to deal with.

In the discrimination study, we found a lower accuracy for the short in comparison to both middle and long vowel durations. That is, more information led to higher response accuracy. The reaction time data revealed that short vowels were responded to more quickly than middle or long vowels. In addition, middle vowels triggered longer reaction times than long vowels. It seems thus that the intermediate category is the hardest one to deal with. Looking at the response times, we further found that participants gave the fastest answers for a StepDistance of 0, followed by StepDistances of 3, 2, and 1. Hence, identical cases posed the smallest challenge to the individuals; beyond that, a greater distance between two sound files of a trial accelerated the reaction.

Further, the interaction GermanPillai × StepDistance reached significance in the accuracy data. Despite the significance, the interaction is neglectable. On the one hand, we saw that /eː/-/ɛː/ maintainers responded slightly more accurately than /eː/-/ɛː/ mergers for a StepDistance of 0; on the other hand, /eː/-/ɛː/ mergers answered slightly more correctly for the StepDistances 1 and 2. StepDistance 3 falls, roughly speaking, in the middle. Overall, the discrimination study can only be interpreted to support H0: /eː/-/ɛː/ maintainers and /eː/-/ɛː/ mergers do not differ with regard to the discrimination of the English vowels /ɛ/ and /æ/.

We realize that sociophonetic aspects of the L1, in our case the degree of overlap of the two German vowels /eː/ and /ɛː/, affect how language users deal with certain phenomena in the L2. Our results provide a piece of evidence that the concept of the L1 and its function in L2 acquisition is heterogenous. Although there is by now a good amount of research targeting different types of sociolinguistic variation in the L2 itself (see Geeslin, 2022 for an overview), we still need more investigations on such variation in the L1 and its impact on L2 acquisition. For instance, it is not clear at all whether speakers of a specific L1 variety have more or fewer difficulties in acquiring a specific L2 than speakers with the same L1 but a different variety of this L1. As we saw in section 1, the vocalic diversity from German we examined in our studies often has a geographical origin. We saw that this vocalic diversity in the L1 translates into patterns in L2 acquisition. Interestingly, we have so far tested participants from one area only (northern Germany, Low German language area), but still find these results. Remember that the two German vowels are often merged in northern Germany, and remember also that variation still exists in all regions. A promising avenue for future research is therefore to take other varieties of German into consideration, which would open at least two additional perspectives. For one, it would be worth comparing speakers from an area where the two vowels /eː/ and /ɛː/ are typically merged, say northern Germany, to those from an area where the two vowels are usually kept apart, for instance, southwestern Germany. Second, we know even of regions in German-speaking Switzerland where [æ] / /æ/ is used as a variant of /ɛ/ (see Kleiner, 2011), that is, a vowel which was new to our participants. They were confronted with it in English but they did not know it from their L1 (German) variety. The question that arises is how German L1 speakers who know [æ] / /æ/ from their L1 would deal with the English vowels in comparison to German L1 speakers who do not know these vowels from their L1.

Finally, we would like to point out another avenue for future research. As we noted in section 1, varieties of English differ with regard to how the phonological contrast between /æ/ and /ɛ/ is articulatorily and acoustically realized. It goes without saying that it is hard to assign an L2 speaker of English to a specific variety since everyone is exposed to several sources of input, each having its distinct nuances. For instance, one might have had a teacher speaking a variety of US American English at school but later lecturers from the United Kingdom at university. To complicate matters even further, learners can be confronted with yet other varieties of English through the media, in international communities, or during extended stays in English-speaking countries. That being said, and although it would not be a trivial task to find L2 speakers who have been exposed to and used a single variety of English only or at least primarily, disentangling the influence of various Englishes would provide us with even more pieces of the puzzle of how individuals acquire speech in an L2.

## 4. Conclusion

Our findings show that sociophonetic variation within a person’s L1 plays a role in L2 acquisition. Individual variation adds an essential piece to the puzzle of how the L1 determines how language users deal with phenomena in the L2. Here, we showed that the degree to which two vowels are merged in someone’s L1, such as /eː/ and /ɛː/ in German, seems to affect the identification of a new vowel (/æ/) in the L2. We feel that there are promising avenues for future research in this field and hope that this work inspires others to enrich our knowledge on the role of individual variation in the interplay between L1 and L2 speech.

## Data availability statement

The R scripts and the datasets are included in the Supplementary material, further inquiries can be directed to the corresponding author.

## Ethics statement

The studies involving human participants were reviewed and approved by Research Ethics Committee, University of Oldenburg, Germany. The participants provided their written informed consent to participate in this study.

## Author contributions

MS and JP developed together the research questions against the background of the relevant literature in the field. MS and MF compiled the materials, designed the experiments, collected the data, conducted the statistical analyses, and wrote the manuscript. All authors contributed to the article and approved the submitted version.

## Conflict of interest

The authors declare that the research was conducted in the absence of any commercial or financial relationships that could be construed as a potential conflict of interest.

## Publisher’s note

All claims expressed in this article are solely those of the authors and do not necessarily represent those of their affiliated organizations, or those of the publisher, the editors and the reviewers. Any product that may be evaluated in this article, or claim that may be made by its manufacturer, is not guaranteed or endorsed by the publisher.

## Footnotes

^1^It should be noted at this point that varieties of English vary in how they implement the contrast between the two vowels acoustically (for discussion, see Sönning, 2020). For instance, while /æ/ is articulated with a higher F1 in (Standard) British English, leading to a greater quality difference between the two vowels in this variety, the duration difference between the two vowels is greater in (Standard) American English (Sönning, 2020). We will come back to this in Section 3.

^2^Please note at this point that we simply continue with the well-known observation that for L1 German speakers German /ɛ/ and /ɛː/ play a decisive role when dealing with English /æ/. Although /ɛ/ and /ɛː/ are close to /æ/, other German vowels might be argued to be close to /æ/, too. Also, individuals with other L1s, which do have /ɛ/ and /ɛː/, too, and which do not have /æ/ either, may rely on other vowel categories in proximity of /æ/, e.g., /a/. The term “differential substitution” has been used to refer to this situation in which the specific substitute for a speech sound not available in someone’s L1 depends on a person’s L1 (see Weinberger, 1988; Brannen, 2011). It is beyond the scope of the present paper to discuss this phenomenon in detail, but we agree with one of the reviewers that it deserves at least a mention here. Defining the concept of proximity when it comes to sound substitutions is a complex field on its own.

^3^From the originally 56 participants, we excluded three who were not from the Low German language area and two who considered themselves to be diverse. “Low German language area” refers to regions in northern Germany where, in addition to Standard German, the regional language of Low German is spoken by some speakers.

^4^We would like to add a note on using a reading task to measure whether the vowels are merged or not. Even though speakers produce a contrast between two vowels in a reading task, one might argue that they do not necessarily articulate this contrast in conversational speech. We still rely on the results from the reading study for two reasons. First, as the results from Elmentaler and Rosenberg (2015) show, for the pronunciation of /ɛː/ no significant differences emerged between reading pronunciation, interviews, and informal conversations among family and friends in northern Germany. Second, the mean Pillai score of the 51 participants was 0.62 (Range: 0.01–0.96; SD: 0.22). That is, we find a good balance between /eː/−/ɛː/ maintainers and /eː/-/ɛː/ mergers in the dataset, rather than a clear tendency toward the maintenance of the contrast.

^5^The tidyverse package (Wickham et al., 2019) was also involved in the statistical analysis.

^6^For binomial logistic regression, we relied on the z statistics.

^7^We used the same ISI throughout the study. For a discussion about how different ISIs affect speech perception (see Werker and Tees, 1984; Werker and Logan, 1985). Future work could possibly implement different ISIs and examine how this affects the participants’ performance.

^8^One might argue that assimilation to German /ɛː/ seems to be the more plausible option for /eː/−/ɛː/ maintainers since they selected the /æ/ word in roughly 60% only. However, we need to take into consideration that long vowel durations (reflecting /æ/) were also crossed with higher steps of our continuum (reflecting /ɛ/). In other words, one acoustic parameter might have canceled out the effect of the other. Therefore, we feel that the findings reported here for vowel duration leave us with two options for /eː/−/ɛː/ maintainers: Either they assimilated English /æ/ to German /ɛː/ or they have developed the new category /æ/.
